# Evaluation of a Point-of-care ultrasound (POCUS) workshop for peripheral intravenous cannulation

**DOI:** 10.1186/s12909-023-04428-5

**Published:** 2023-06-19

**Authors:** Ulrich Steinwandel, Linda L. Coventry, Homa Kheirkhah

**Affiliations:** 1 School of Nursing and Midwifery, Edith Cowan University, 270 Joondalup Drive, Joondalup, 6027 WA Australia; 2 Centre for Research in Aged Care, Edith Cowan University, Joondalup, Australia; 3 Centre for Nursing Research, Sir Charles Gairdner Hospital, Nedlands, Australia

**Keywords:** Cannulation training, Medical education, Peripheral intravenous cannulas, Peripheral intravenous catheters, PIVC, POCUS, Point-of-care ultrasound, Simulation training, Vascular access, Ultrasound

## Abstract

**Background:**

Point-of-care ultrasound (POCUS) is increasingly used as a non-invasive vascular access assessment method by clinicians from multiple disciplines worldwide, prior and during vascular access cannulations. While POCUS is a relatively new method to establish a vascular access in patients with complex vascular conditions, it is also essential to train and educate individuals who are novices in the techniques of cannulation so that they become proficient in performing this task subsequently on patients safely and successfully. A simulated environment may be a helpful tool to help healthcare providers establish skills in using POCUS safely and may also help them to successfully establish vascular access in patients. With this project, we sought to determine if participants of a simulated POCUS workshop for vascular access can use this technique successfully in their individual clinical environment after their attendance of a half-day workshop.

**Methods:**

A mixed-methods longitudinal study design was chosen to evaluate a point-of-care ultrasound workshop for peripheral intravenous cannula insertion. The workshops used simulation models for cannulation in combination with multiple ultrasound devices from various manufacturers to expose participants to a broader variety of POCUS devices as they may also vary in different clinical areas. Participants self-assessed their cannulation skills using questionnaires on a 10-point rating scale prior to and directly after the workshop.

**Results:**

A total of 85 Individuals participated in eleven half-day workshops through 2021 and 2022. Workshop participants claimed that attending the workshop had significantly enhanced their clinical skill of using ultrasound for the purpose of cannulating a venous vessel. The level of confidence in using this technique had increased in all participants directly after conclusion of the workshop.

**Conclusions:**

Globally, clinicians are increasingly using POCUS to establish vascular access in patients, and it is necessary that they receive sufficient and adequately structured and formal training to successfully apply this technique in their clinical practice. Offering a workshop which uses simulation models in combination with various POCUS devices to demonstrate this technique in a hands-on approach has proven to be useful to establish this newly learned skill in clinicians.

**Supplementary Information:**

The online version contains supplementary material available at 10.1186/s12909-023-04428-5.

## Background

The use of point-of-care ultrasound (POCUS) for vascular access cannulation has been previously described as a useful skill to establish a vascular access for haemodialysis patients [1–3]. Other clinical specialities such as emergency departments have also successfully adopted this method of using POCUS to insert peripheral intravenous catheters (PIVCs) in patients with difficult venous access (DIVA) [4, 5]. Furthermore, the insertion of a PIVC has also been previously described as the most common invasive procedure in hospital care worldwide [6, 7]. It has also been described, that success rates with ultrasound-guided PIVC insertions usually improve the more often such a procedure is performed [8]. As this task is most often performed by either nurses or physicians [9, 10], these healthcare providers need, first and foremost, appropriate training to be successful with the technique of utilising POCUS when cannulating patients with DIVA.

POCUS devices today are becoming more and more affordable, portable, and versatile due to wireless functionality and becoming increasingly available in all clinical areas in Australia, with some devices’ price tags ranging currently from ‘as little as’ $6,000 Australian Dollars for the Philips Lumify (Philips, Best, Netherlands) to around $7,000 Australian Dollars (Vscan Air – GE Healthcare, Chicago, Illinois) for wireless POCUS devices. It is therefore obvious, that clinicians may want to use those, now more affordable devices in their clinical practice, as it may help and support them to navigate and manage a DIVA cannulation. Additionally, there is today also a broader variety of different POCUS devices available in Australian healthcare facilities compared to the past.

Local guidelines have recommended to use ultrasound devices to guide the insertion of the PIVC [11] and it was also suggested that educational programs are developed for junior doctors to support their safe and appropriate use [12]. The same researchers also concluded that individuals who received formal face-to-face teaching on using ultrasound were more successful in establishing vascular access compared to individuals who did not receive any education [12]. A simulation based PIVC insertion course in the United States (US) that combined online and simulated based instructions revealed a significant improvement in nurses’ knowledge, confidence, and skills for PIVC insertion in a simulated environment which resulted in fewer complications and enhanced patient outcomes [13]. Archer-Jones, Sweeny [4] performed a study evaluating an ultrasound-guided peripheral intravenous training program for emergency clinicians such as nurses and doctors and reported that this training increased confidence in the cannulation task itself as well as in using ultrasound for the purpose of achieving improved success with cannulations in the participants. A POCUS training program in the United States (US) revealed that physicians can gain POCUS skills through a brief, 2.5-day training course [14] and these individuals were able to retain the attained knowledge eight months after the short-term course. Specific healthcare professions, such as haemodialysis nurses, may generally receive some formal training on the cannulation of vascular access without the use of ultrasound as it is part of their daily routine [15]. The American Institute of Ultrasound Medicine states, “that there are no absolute contraindications to using ultrasound as a procedural adjunct for vascular guidance” [16]. Additionally, a recent Dutch study on the likelihood of difficult intravenous access has recommended that POCUS can be indicated for ‘at-risk’ DIVA patients [17]. An American study reported that DIVA is a common issue in Emergency Departments (EDs) and when nurses were appropriately trained in using POCUS for the insertion of PIVCs, it may subsequently improve the care for patients with DIVA [18].

There is limited research on DIVA patients and POCUS related education in Australia. To date, there has been only one pilot study performed in Victoria (Australia) describing the success of an educational intervention using POCUS and phantom models in connection with peripheral intravenous access for intensive care paramedics [19].We could not identify any further previous research describing the POCUS education of other novices such as medical doctors or nurses and also not for our local setting in Western Australia (WA). Although more POCUS devices may exist in the clinical area today. So far, we know of only some anecdotal evidence that a few clinicians provide training in the use of POCUS for novices in WA. Additionally, we could not identify any previous research in the space of POCUS training, which had documented the various educational stages when participants were taking up a simulative approach to learn and apply this technique.

It has been previously described that a simulative environment helps students to learn through an interactive element [20], but we could not identify scientific literature which had documented an individual’s learning progress during the different stages of the workshop.

This research project aimed to evaluate the experience of Western Australian health care professionals attending a half-day workshop which teaches the practical skills of ultrasound guided cannulation using simulation models and POCUS devices in inserting PIVCs. We also aimed to measure the satisfaction level of workshop participants with the overall workshop presentation and potential usefulness of the learned content for their individual clinical practice.

## Methods

### Study design

We used an observational longitudinal mixed methods study approach using electronic surveys to assess the participants’ confidence level using POCUS during a peripheral venous cannulation.

### Participants

Participants included enrolled nurses, registered nurses, clinical nurse consultants, clinical nurse specialists, staff development nurses, resident medical officers (junior doctors), dental sedationists, nuclear medicine technicians and diagnostic radiologists from local and regional public and private healthcare institutions.

### Workshops

A series of 11 single face-to-face workshops over a period of 13 months between July 2021 and August 2022 were performed at the Edith Cowan University campus, School of Nursing and Midwifery / Joondalup, Western Australia with the number of participants ranging from four to 14 in each session. One session was performed externally at a local satellite dialysis clinic in regional Western Australia (WA).

A half-day workshop was designed to teach ultrasound-naïve healthcare professionals the practical skill of using ultrasound prior and during the cannulation of a peripheral venous blood vessel. This half-day workshop consisted of four hours of educational content, the first hour of the workshop comprising the theoretical background of using ultrasound for the purpose of vascular access cannulation and the basic principles of identifying suitable blood vessels, as well as how to interpret observations made using a POCUS device. Infection control principles and aseptic non-touch technique (ANTT) were also key components of the interactive presentation. In the second hour, workshop participants were invited to observe the demonstration of vascular access cannulation in the simulation model, using POCUS by the workshop facilitator. For this purpose, simulation models created from chicken breast and fluid filled modelling balloons were used to simulate human tissue and human blood vessels as previously suggested by Rippey, Blanco [21]. The third and fourth hour of the workshop were reserved for hands-on practice of the newly adopted skill wherein participants were offered guidance while cannulating the chicken breast phantom models using a variety of POCUS devices. Three different portable and cart-based POCUS devices from a variety of manufacturers were used, such as the Philips Lumify (Philips, Best, Netherlands), the Sonosite Edge II (Fujifilm) and Vscan Air (GE Healthcare, Chicago, Illinois) device, which were in some instances also available in some participants’ workplace. Different POCUS devices were used as devices may vary in different clinical areas in various healthcare institutions. The workshops were delivered with a facilitator to participant ratio varying from 1:4 up to 1:13 for all workshops during the duration of the study.

### Data collection

Workshop participants were invited to complete an initial survey prior to the attendance of the workshop which assessed practical clinical cannulation skill; workplace location; years of clinical experience; gender; clinical position; age; the availability of a POCUS device and if they had used it previously. All workshop surveys were developed by the Principal Investigators. The initial self-assessment from respondents regarding their current skill level in using ultrasound while cannulating was rated on a 10-point rating scale (0 = not very skilled to 10 = very proficient). Additionally, participants were asked, if they had previously observed colleagues using POCUS in combination with DIVA and if their employer would pay for the workshop participation. Participants could also indicate any previous experiences of observing if competent others were using POCUS in their clinical setting. See Supplementary Material, Additional file 1 ‘Qualtrics Survey Before ultrasound workshop.pdf’ A second survey was provided to participants directly after conclusion of the workshop, with the intention to record any newly achieved POCUS skill level, again on a self-assessment scale. This second survey consisted of 11 closed and three open-ended questions. Again, they were invited to rate their current individual skill in using ultrasound while cannulating, again rated on a 10-point rating scale (0 = not very skilled and 10 = very proficient). See Supplementary Material, Additional file 2 ‘Qualtrics Survey After ultrasound workshop’. Eight weeks after the completion of the workshop, participants were electronically sent a final survey which invited them to record any further progress in adopting and applying the new skill in their clinical workplace. This survey also asked for a self-assessment of the skill of USGC. This last survey comprised nine closed and two open-ended questions. See Supplementary Material, Additional file 3 ‘Qualtrics Survey 8 weeks After workshop.pdf’.

### Data analysis

Descriptive statistics were analysed using IBM SPSS statistics (SPSS version 28, IBM, SPSS Inc.). Figures were presented using excel (Microsoft Corporation. Microsoft Excel [Internet]0.2018). Self-assessed cannulation skills over the three time periods were analysed using a Friedman Test and post-hoc analysis was conducted using the Wilcoxon sign-ranked tests with a Bonferroni correction applied. A p-value < 0.05 was considered statistically significant. Content analysis was performed for the open-ended questions.

### Ethical considerations

At workshop commencement, workshop participants were informed (verbally and in writing) about the research project using a participant information letter and were also given ample time to read this information and had also the opportunity to ask questions. Participant consent to participate in the surveys was assumed when participants completed the survey. Survey data were stored and analysed on a password protected university owned computer with only the chief investigator and the associate investigator having access to the data.

This study was approved by the Edith Cowan Universities Human Research Ethics committee under the Reference number REMS 2021–02489-STEINWANDEL. Before and after-workshop surveys were conducted using electronic tablet devices (Samsung Galaxy Tab) and including QR codes which then directed participants to an online survey platform (Qualtrics, Provo, Utah, United States).

## Results

A total of 85 healthcare professionals participated in 11 workshops with two thirds of the participants female (*n* = 55, 67.9%), with a mean age of 35.7 years. The mean years of clinical experience was eight years with almost half (*n* = 40, 49%) with two years or less clinical experience. The majority of participants were resident medical officers (*n* = 43, 53%), followed by clinical nurses / staff development / clinical nurse consultants (*n* = 19, 25.3%). Other participants included a Nuclear Medicine Technologist, a Dental Sedationist and a Diagnostic Radiologist. Most participants (*n* = 68, 84%) were employed at public hospitals. Almost a quarter (*n* = 20, 23.5%) were employed at haemodialysis (HD) clinics which were either located in a regional hospital or in a satellite haemodialysis clinic in Western Australia. Of these, the clinical experience working in the field of HD ranged from 1 to 30 years. Almost all participants had ultrasound devices available in their workplace (*n* = 75, 93.8%); most had never used an ultrasound to cannulate (*n* = 47, 67.1%); however, the majority had been shown by their colleagues how to use an ultrasound (*n* = 39, 56.5%) and had observed colleagues using ultrasound to cannulate (*n* = 62, 88.6%). Baseline demographics are presented in Table 1.

**Table 1:** Baseline demographics

Variable	
Age, years	*N* = 81
Mean (SD)	35.7 ± 11.7
Median (IQR)	32.0 (26.0, 45.0)
Years of clinical experience	*N* = 81
Mean (SD)	8.4 ± 10.5
Median (IQR)	3.0 (1.0, 15.0)
Range	0.0 to 45.0
	*n* (%)
Gender	*N* = 81
Female	55 (67.9)
Male	26 (32.1)
Prefer to not say	0 (0.0)
Current position	*N* = 75
Enrolled nurse	2 (2.7)
Registered nurse	14 (18.7)
Clinical nurse / Staff Development Nurse / Clinical Nurse Consultant	19 (25.3)
Resident medical officer (RMO)	38 (50.7)
Other	2 (2.7)
Work setting	*N* = 81
Public hospital	68 (84.0)
Private health care institution	13 (16.0)
Ultrasound device available	*N* = 81
Yes	75 (93.8)
No	4 (5.0)
No, but it is intended to acquire an U/S	1 (1.3)
Have you previously used ultrasound to cannulate?	*N* = 70
Never	47 (67.1)
Seldom	12 (17.1)
Sometimes	8 (11.4)
Usually	3 (4.3)
Always	0 (0.0)
Have your colleagues shown you how to use ultrasound?	*N* = 69
Yes	39 (56.5)
No	30 (43.5)
Have you observed colleagues using ultrasound for cannulations?	*N* = 70
Yes	62 (88.6)
No	8 (11.4)

Post workshop evaluation was completed by 81 (95%) participants. Eight weeks after the workshop, participants, who indicated in the post-workshop survey to be contacted again, were invited via email to respond to a final survey which aimed to record their individual progress in adopting this new technology in their clinical practice. Just over a half of the participants (*n* = 43, 53%) responded to this last and final survey. At post workshop evaluation and eight weeks after workshop evaluation,. almost all participants planned on using ultrasound more frequently (post workshop, *n* = 78, 98%; and 8-weeks post, *n* = 42, 98%) and almost all agreed participating in the workshop was useful for their personal development (post workshop, *n* = 79, 99%; 8-weeks post, *n* = 43, 100%). Additionally, almost two thirds thought their workplace would allow extra time to practice this new skill (post workshop, *n* = 57, 71%; 8-weeks post, *n* = *n* = 27, 63%). At 8-weeks post workshop most stated, they would appreciate if there would be a colleague or an educator at their workplace available, who would be able to guide them further with the interpretation of the observed images (*n* = 35, 81%). See Table 2

**Table 2:** Post workshop evaluation

Variable	Total N	Strongly disagree n (%)	Disagree n (%)	Neither n (%)	Agree n (%)	Strongly agree n (%)
**Post workshop evaluation**						
I plan on using ultrasound more frequently	80	2 (2.5)	0 (0.0)	0 (0.0)	23 (28.8)	55 (68.8)
Participating in this workshop has been useful for my professional development?	80	1 (1.3)	0 (0.0)	0 (0.0)	19 (23.8)	60 (75.0)
Will your workplace allow the additional time you will need to use for practicing and refining this new skill?	80	1 (1.3)	6 (7.5)	16 (20.0)	39 (48.8)	18 (22.5)
This workshop met my learning needs in relation to using and applying POCUS in my clinical practice	80	2 (2.5)	0 (0.0)	0 (0.0)	32 (40.0)	46 (57.5)
**Eight weeks after workshop evaluation**						
I plan on using ultrasound more frequently	43	1 (2.3)	0 (0.0)	0 (0.0)	25 (58.1)	17 (39.5)
An on-site colleague / educator would be helpful to guide me further with the interpretation of the observed images	43	0 (0.0)	2 (4.7)	6 (14.0)	24 (55.8)	11 (25.6)
Participating in this workshop has been useful for my own professional development?	43	0 (0.0)	0 (0.0)	0 (0.0)	24 (55.8)	19 (44.2)
Will your workplace allow the additional time you will need to use for practicing and refining this new skill?	43	0 (0.0)	3 (7.0)	13 (30.2)	21 (48.8)	6 (14.0)

Workshop participants were asked what the best aspects of the workshop were and how they would rate the knowledge and presentation skills of the workshop facilitator. Over 60 encouraging comments were made, with themes predominantly regarding the nature of the practical hands-on experience for workshop participants in this setting, the opportunity to be observed by the facilitator while practicing a newly adopted clinical skill and the connection between theory and practice, and finally the opportunity to spending plenty of time in the use of new technology, which aims to improve patient care. Many individuals (*n* = 71, 88%) rated the knowledge of the workshop facilitator as ‘excellent’ and most participants (*n* = 68, 84%) regarded his presentation skills as ‘excellent’. Data not presented in tables.

**Fig. 1:**
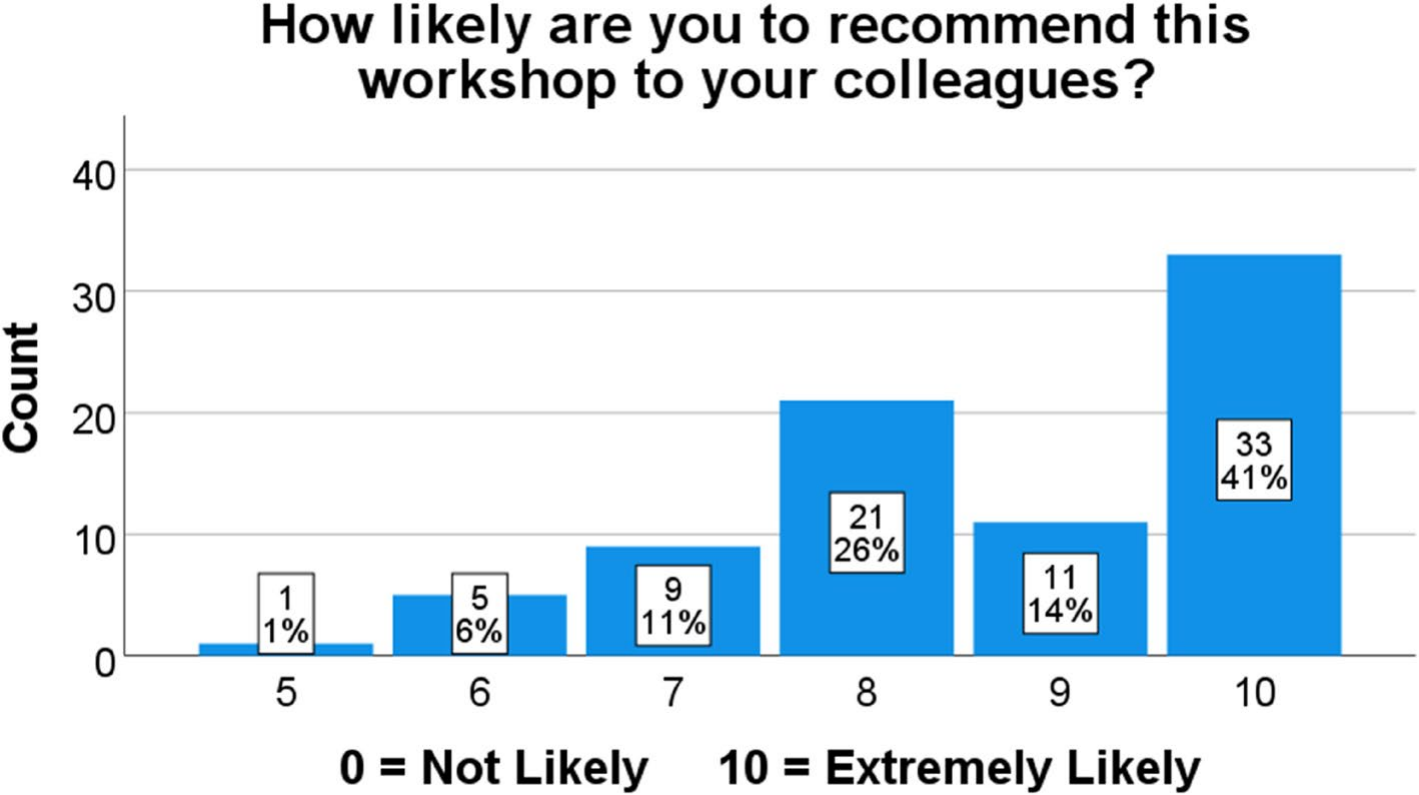
Level of recommendation of workshop provided by participants upon workshop conclusion

Participants were further asked if they would recommend attendance of the workshop to their colleagues. A numeric scale with a rating ranging from 0 (Not at all likely) to 10 ( Extremely likely) for the recommendation of this workshop to others was used. The majority scored 10 and were extremely likely to recommend this workshop (*n* = 33, 41%). See Fig. 1.

Eight weeks after the workshop, almost one third had time to practice this new skill (*n* = 14, 32.6%); however, most of the participants had very little time to practice (*n* = 16, 37.2%). An equivocal number of participants had time to demonstrate this skill to their colleagues (*n* = 17, 39.5%) while other participants did not have time demonstrate this skill to others (*n* = 16, 37.2%). A few (*n* = 7,16.3%) had no access to an ultrasound device at their current workplace. See Table 3.

**Table 3:** Eight weeks after workshop evaluation

Variable	*n* (%)
Have you had time to practice this new skill in your own work environment?	*N* = 43
Definitely yes	14 (32.6)
Only very little	16 (37.2)
I would have loved to practice but I had no access to an ultrasound device	7 (16.3)
I am planning on practicing this skill more frequently	3 (7.0)
Definitely not	3 (7.0)
Have you had time to show and demonstrate this new skill to a work colleague?	*N* = 43
Definitely yes	17 (39.5)
Only once	10 (23.3)
Definitely not	16 (37.2)

When asked, what further measures would be required to establish using ultrasound at their workplace, just under a half (*n* = 18, 42%) stated, that they would need at least one or more portable ultrasound devices being made available in their clinical setting to have more practice opportunities. Some individuals stated that a clinical competency pathway in using POCUS in connection with DIVA should be established and implemented in their department. One individual also reported that some senior doctors were reluctant to allow nurses to use this novel approach and that in their Emergency Department, nurses needed to perform and demonstrate a minimum of four successful POCUS cannulations, observed by a POCUS trained doctor to be deemed competent in this technique.

**Fig. 2:**
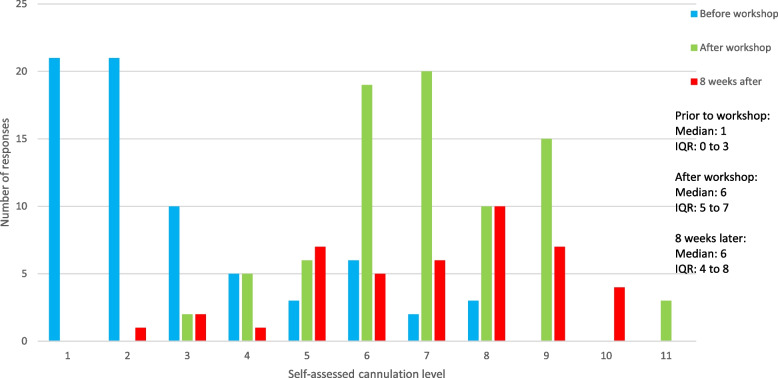
Level of self-assessed cannulation skill level provided by participants before, directly after (on conclusion) and eight weeks after the workshop

Self-assessed cannulation using POCUS before, directly after and 8-weeks after the workshop determined by participants is graphically displayed in Fig. 2. There was a statistically significant difference in self-assessed cannulation skill over the three time periods (*p* < 0.001). Post hoc analysis revealed a statistical difference in self-assessed cannulation skill before the workshop compared with after the workshop (*p* < 0.001); and self-assessed cannulation skill before the workshop compared with 8 weeks after the workshop (*p* < 0.001). There were no differences in self-assessed cannulation skill immediately after the workshop compared with 8-week after the workshop (*p* = 0.722). p-values are presented in Table 4.

**Table 4:** *p*-values of self-assessed cannulation skills by participants before, directly after and eight weeks post workshop

	Chi-square	Degrees of freedom	Z score	p-value
Self-assessed cannulation skills before, after and 8-weeks after workshop	52.738	2		< 0.001^1^
Cannulation self-rating before workshop, compared to cannulation self-rating after workshop			7.131	< 0.001^2^
Cannulation self-rating before workshop, compared to cannulation self-rating eight weeks after workshop			5.416	< 0.001^2^
Cannulation self-rating after course, compared to cannulation self-rating eight weeks after course			0.355	0.722^2^

^1^Friedman test; ^2^Wilcoxon Signed-Rank Test

## Discussion

Although there have been previously guidelines developed for the use of ultrasound guiding vascular access procedures, such as the by the American Institute of Ultrasound in Medicine (AIUM) [22] and also the current ‘Management of peripheral Intravenous catheters – Clinical Care Standard’ published by the Australian Commission on Safety and Quality in Health Care from May 2021, which states that ‘the use of advanced techniques such as ultrasound are often needed’, they may still not be part of the practical training of healthcare professionals of various professions. Some individuals from the United Kingdom have recommended the use of POCUS to be added to the curriculum of teaching medical students as it’requires minimal training to operate, can be quickly performed and easily supervised and also later supervised, if scans are saved’ [23]. The use of POCUS in connection with DIVA has been proven to increase cannulation success rates and may also reduce the dependence on senior or more experienced doctors [24].The increased practical use of POCUS is not just happening in the medical workforce but becomes also more prevalent amongst nurses and nurse practitioners [25] and also renal nurses [2, 3]. It is therefore necessary to train and educate novices using this technique through theoretical and practical demonstrations which include subsequent individual hands-on experience for participants to individually learn and perform this skill. Some junior doctors participating in our study reported, that they received only 30 min practical vascular access cannulation training previously by their medical school before they entered the clinical setting. Our study has shown that the participants were satisfied with this educational approach and also that through previous work by others, a structured approach in teaching this technique may be a useful method to introduce the use of POCUS in connection with vascular access cannulation to establish this technique in clinical practice in Australia.

### Strengths and limitations

To our knowledge, this is the first study which investigates the impact of an ultrasound guided workshop on the subsequent clinical practice of predominantly junior clinicians when they are challenged by DIVA conditions in patients in WA. The findings of this study may only be applicable in the educational context of clinicians located in Western Australia and may not be generalisable to other geographical regions. The participants who completed the 8-week follow-up survey were more likely to be motivated individuals who were going to use POCUS and were enthusiastic and likely to answer the survey, therefore this may have biased the results. Our study utilized a newly developed scale as a main outcome measure. Further studies are needed to validate the utility of this tool for future implementation and research. Also, additional increased uptake of POCUS in connection with DIVA in future may result in more skilled individuals who may be able to share their practical skills with other healthcare professionals on a local level. Additionally, different workshop designs and/or structures in future may result in different learning outcomes for participants. Also, workshops with a longer duration (full days or several consecutive days) or including a defined follow-up period where learners can be observed subsequently in their clinical setting by a facilitator may also lead to different levels of competency in individuals. Future research studies should address these potential differences.

## Conclusion

Our study has demonstrated, that using a simulative approach in teaching the skill of cannulation in connection with POCUS increases the skill level from participants over the course of the workshop and afterwards. A few participants were also able to use the newly learned skill at their workplace and all participants found this educational activity useful for their own professional development and would therefore recommend participation. This educational activity may also be beneficial to increase the uptake of this new approach by clinicians when DIVA conditions in patients prevail, as some workshop participants may want to share their experience with others. Some workshop participants indicated that more POCUS devices could be helpful in their clinical setting and that they would appreciate a clinical educator, or an instructor on-site, which would potentially help them to refine their practical skills when cannulating with POCUS. The large proportion of junior doctors (more than half) amongst all participants is also reflective of the clear need for advanced education in the setting of frontline workers being confronted with DIVA conditions when they are caring for patients. Additionally, it may also be useful to create and integrate clinical competency pathways and clinical guidelines for the use of POCUS in combination with vascular access cannulation.

### Implications for practice

This study demonstrates the need for ongoing comprehensive and up-to-date practical hands-on education in the use of POCUS in connection with DIVA, especially for novice clinicians who want to use this technique.

## Supplementary Information

**Additional file 1. **Qualtrics Survey Before ultrasound workshop.pdf

**Additional file 2. **Qualtrics Survey After ultrasound workshop.pdf

**Additional file 3. **Eight weeks after workshop survey (distributed via email) – Qualtrics Survey 8 weeks After ultrasound workshop via email.pdf

## Data Availability

The datasets used and/or analysed during the current study are available from the corresponding author on reasonable request.
