# Supplementary material for: Evaluation of a Point-of-care ultrasound (POCUS) workshop for peripheral intravenous cannulation
Source: BMC Med Educ. 2023 Jun 19;23:451. doi: 10.1186/s12909-023-04428-5 (PMC10280877; doi:10.1186/s12909-023-04428-5)
Supplement: Supplementary file 1 — Additional file 1. Qualtrics Survey Before ultrasound workshop.pdf [file 12909_2023_4428_MOESM1_ESM.pdf]

Block 1

Dr Ulrich Steinwandel

School of Nursing and Midwifery

Edith Cowan University

270 Joondalup Drive

JOONDALUP WA 6027

Phone: 6304 5177

Email: u.steinwandel@ecu.edu.au

Project Name: Ultrasound guided cannulation workshop evaluation
Information Letter to Participants
We are conducting a study to explore the learning outcomes of renal nurses attending a half-day practical educational workshop in utilizing a portable ultrasound device prior to the cannulation of a vascular access of patients on haemodialysis using phantom models. This study is being undertaken through the School of Nursing and Midwifery at Edith Cowan University. The researcher conducting this study is Dr Ulrich Steinwandel. The following will explain to you all procedures involved in the project before you decide whether or not to participate in this study. Please read the information carefully.
Why were you chosen for this research?
You have been invited to participate in this survey, as you are a Registered or Enrolled Nurse involved in cannulation the vascular access of haemodialysis patients and you are attending this workshop which aims to teach you the skill of

ultrasound guided cannulation.
What are the benefits to you?
There will be no direct benefit to you for participating in this study, but indirectly your responses have the potential to inform clinical practice which may contribute to improved health outcomes for patients in the future. The findings of this study will identify how much an educational activity, as this workshop, can improve the practical clinical skills of renal nurses and also, if such a workshop may be potentially useful for other nursing professions.
What does the research involve?
- An anonymous questionnaire that will take approximately 5 minutes to complete prior to the workshop
- A second questionnaire at conclusion of the workshop, which will also take approximately 5 minutes to complete
- Inconvenience in participating in this research is expected to only be that of your time
- Your participation is voluntary and if you decide to complete the survey, we assume you were giving your verbal consent to participate in this study
- You can withdraw from the study at any time by exiting the survey before you submit your answers (It will not be possible to withdraw once answers are submitted as your responses are anonymous)
- It is your choice, whether or not to take part in this study, there will be no potential disadvantages for you resulting from your decision. If you decide you do not want to take part, it will not affect your learning experience in this workshop.

Confidentiality & Storage of Data
Data from the survey will only be accessible by the research team. Electronic data will be password protected and hardcopies stored in accordance with ECU regulations. At the end of seven years, data related to the study will be destroyed. A report of the study will be submitted for publication allowing open access to data from the report to be shared with like-minded researchers, but individual study participants will not be identifiable in such a report.

Questions and/or further information
If you as a participant have any questions, require any further information or would like to learn of the results, please contact Dr. Ulrich Steinwandel: u.steinwandel@ecu.edu.au

Independent contact person
If you have any concerns or complaints about the research project and wish to an independent person, you may contact:

Research Ethics Officer
Edith Cowan University
270 Joondalup WA 6027
Phone: (08) 6304 2170 Email: ethics@ecu.edu.au

Workshop evaluation

I have been provided with the Participant Information Sheet explaining the research study
- I have read and understood the information provided
- I have been given the opportunity to ask questions and have had any questions answered to my satisfaction
- I understand that participation is voluntary and I can withdraw at any time before completing the survey by contacting the Lead Researcher
- I understand that the information provided will only be used for the purposes of this research project
- I understand that the findings of this study may be published but will not include any identifying information I acknowledge the above statements and agree to participate in this study

- Yes
- No

Please generate a unique participant identifier by combining your mother's first given name and your (full) year of birth (e.g. Caroline1968).
Please remember this identifier for the second questionnaire (after completion of the workshop).

On a scale from 1 to 10 how would you rate your current practical clinical cannulation skills using portable ultrasound (POCUS)? (With 10 being 'very proficient' and 1 being 'not very skilled'

	0	1	2	3	4	5	6	7	8	9	10
current practical cannulation skills											

Please indicate your workplace location?

- satellite haemodialysis clinic
- major tertiary hospital in Perth metro
- hospital
- WACHS

Are you working in a ...?

- public hospital
- private healthcare institution

Overall, how many years of clinical experience do you have? (just enter a number)

How many years of haemodialysis nursing experience do you have? (just enter a number)

Please indicate your gender?

- female
- male
- Prefer not to say

Please indicate your current position

- Enrolled Nurse (EN)
- Registered Nurse (RN)
- Clinical Nurse (CN) / Staff Development Nurse (SDN)
- Clinical Nurse Consultant (CNC) /Clinical Nurse Manager (CNM)
- other

How old are you? just enter a number

Does your unit / workplace own an ultrasound device?

- yes
- no
- no, but it is intended that we acquire one

Have you previously used ultrasound prior to cannulations?

- always
- usually
- sometimes
- seldom
- never

Have colleagues shown you how to use ultrasound?

- yes
- no

Have you observed colleagues using ultrasound before or during vascular access cannulations?

- yes
- no

Will your employer pay for your participation in this workshop?

- No, but I will claim it on tax
- Yes
- I don't know

Participating in this workshop will be useful for my professional development

strongly agree	agree	neither	disagree	strongly disagree
○	○	○	○	○

Powered by Qualtrics
