# Supplementary material for: Evaluation of a Point-of-care ultrasound (POCUS) workshop for peripheral intravenous cannulation
Source: BMC Med Educ. 2023 Jun 19;23:451. doi: 10.1186/s12909-023-04428-5 (PMC10280877; doi:10.1186/s12909-023-04428-5)
Supplement: Supplementary file 2 — Additional file 2. Qualtrics Survey After ultrasound workshop.pdf [file 12909_2023_4428_MOESM2_ESM.pdf]

Workshop evaluation

I have been provided with the Participant Information Sheet explaining the research study
- I have read and understood the information provided
- I have been given the opportunity to ask questions and have had any questions answered to my satisfaction
- I understand that participation is voluntary and I can withdraw at any time before completing the survey by contacting the Lead Researcher
- I understand that the information provided will only be used for the purposes of this research project
- I understand that the findings of this study may be published but will not include any identifying information I acknowledge the above statements and agree to participate in this study

- ○ Yes
- ○ No

Please indicate your self-generated unique identifier again using your mother's first given name and your (full) year of birth (e.g. Caroline 1968)

[ ]

After attending this workshop, and on a scale from 1 to 10 how would you rate now your practical clinical cannulation skills using portable ultrasound (POCUS)? (With 10 being 'very proficient' and 1 being 'not very skilled'

	0	1	2	3	4	5	6	7	8	9	10
cannulation skills using ultrasound											

I am now planning on using this technique more frequently to become more proficient?

strongly agree	agree	neither agree or disagree	disagree	strongly disagree
O	O	O	O	O

Participating in this workshop has been useful for my own professional development?

strongly agree	agree	neither agree or disagree	disagree	strongly disagree
O	O	O	O	O

How likely are you to recommend this workshop to friends or colleagues?

Not at all likely ... Extremely likely

0	1	2	3	4	5	6	7	8	9	10
O	O	O	O	O	O	O	O	O	O	O

Do you think, your workplace will allow for the additional time you will need to use for practicing and refining this new skill?

strongly agree	agree	neither agree nor disagree	disagree	strongly disagree
O	O	O	O	O

This workshop met my learning needs in relation to using and applying POCUS in my clinical practice

strongly agree	agree	neither agree nor disagree	disagree	strongly disagree
O	O	O	O	O

Do you have any comments on how we could improve this workshop or what you would have liked to have more explored / time to practice?

What were the best aspects of the workshop?

How would you rate the knowledge of the workshop facilitator?

Excellent	Good	Average	Fair	Poor
○	○	○	○	○

How would you rate the presentation skills of the workshop facilitator?

Excellent	Good	Average	Fair	Poor
○	○	○	○	○

Do you consent that we contact you in a few weeks via email to see if you had the chance to further advance and practice this skill of using ultrasound?

○ Yes

○ No

If you have consented that we contact you in a few weeks, please provide your e-mail contact below

email

Powered by Qualtrics
