# Supplementary material for: Evaluation of a Point-of-care ultrasound (POCUS) workshop for peripheral intravenous cannulation
Source: BMC Med Educ. 2023 Jun 19;23:451. doi: 10.1186/s12909-023-04428-5 (PMC10280877; doi:10.1186/s12909-023-04428-5)
Supplement: Supplementary file 3 — Additional file 3. Eight weeks after workshop survey (distributed via email) – Qualtrics Survey 8 weeks After ultrasound workshop via email.pdf [file 12909_2023_4428_MOESM3_ESM.pdf]

Workshop evaluation

Dr Ulrich Steinwandel

School of Nursing and Midwifery

Edith Cowan University

270 Joondalup Drive

JOONDALUP WA 6027

Phone: 6304 5177

Email: u.steinwandel@ecu.edu.au

Project Name: Ultrasound guided cannulation workshop evaluation
Information Letter to Participants

Dear workshop participant,

Thank you for previously attending the workshop "Using ultrasound to guide cannulation of the difficult venous access" at ECU. This is just a brief follow-up questionnaire which has been sent to your e-mail address as you have previously agreed to be contacted sometime after the workshop has occurred.

We would therefore like to know a little bit about your progress in using this new technique in your own clinical environment and have therefore attached a few more questions, which we kindly invite you to complete.

It may not take much longer than five minutes for you to complete these questions.

Thank you very much for your participation!

If you have any further queries about this workshop or about this survey, then please contact me under:

Dr. Ulrich Steinwandel

School of Nursing and Midwifery

Edith Cowan University

270 Joondalup Drive

JOONDALUP WA 6027

Phone:6304 5177

Email: u.steinwandel@ecu.edu.au

Questions and/or further information
If you as a participant have any questions, require any further information or would like to learn of the results, please contact Dr. Ulrich Steinwandel: u.steinwandel@ecu.edu.au

Independent contact person
If you have any concerns or complaints about the research project and wish to an independent person, you may contact:

Research Ethics Officer
Edith Cowan University
270 Joondalup WA 6027
Phone: (08) 6304 2170 Email: ethics@ecu.edu.au

Please indicate your previously self-generated unique identifier again, using your mother's first given name and your (full) year of birth (e.g. Caroline 1968)

After attending this workshop, and on a scale from 1 to 10 how would you rate now your practical clinical cannulation skills using portable ultrasound (POCUS)? (With 10 being 'very proficient' and 1 being 'not very skilled'

0 1 2 3 4 5 6 7 8 9 10

	0	1	2	3	4	5	6	7	8	9	10	
cannulation skills using ultrasound												

Have you had time to practice this new skill in your own work environment?

- Definitely yes
- only very little
- I would have loved to practice at my workplace, but I had no access to an ultrasound device
- I am planning on practising this skill more frequently
- Definitely not

I am now planning on using this technique more frequently to become more proficient

- strongly agree
- agree
- neither agree nor disagree
- disagree
- strongly disagree

Do you feel participating in this workshop has been useful for your own professional development?

- strongly agree
- agree
- neither agree nor disagree
- Click to write Choice 6
- disagree

How likely are you to recommend this workshop to friends or colleagues?

Not at all likely										Extremely likely
0	1	2	3	4	5	6	7	8	9	10

Do you think, your workplace will allow for the additional time you will need to use for practicing and refining this new skill?

strongly agree	agree	neither agree nor disagree	disgaree	strongly disagree

Is there anything else you would require to use and implement this new technique in your workplace?

Do you have any comments on how we could improve this workshop or what you would have liked to have more explored / time to practice?

Thank you very much for completing this survey!

Powered by Qualtrics
